# Population Dynamics of Owned, Free-Roaming Dogs: Implications for Rabies Control

**DOI:** 10.1371/journal.pntd.0004177

**Published:** 2015-11-06

**Authors:** Anne Conan, Oluyemisi Akerele, Greg Simpson, Bjorn Reininghaus, Jacques van Rooyen, Darryn Knobel

**Affiliations:** 1 Department of Veterinary Tropical Diseases, Faculty of Veterinary Science, University of Pretoria, Pretoria, South Africa; 2 Gauteng Department of Agriculture and Rural Development, Gauteng, South Africa; 3 Centre for Veterinary Wildlife Studies, Faculty of Veterinary Studies, University of Pretoria, Pretoria, South Africa; 4 Mpumalanga Veterinary Services, Department of Agriculture, Rural Development, Land and Environmental Affairs, Thulamahashe, South Africa; 5 Center for Conservation Medicine and Ecosystem Health, Ross University School of Veterinary Medicine, Basseterre, St. Kitts; Atlanta Health Associates, Inc., UNITED STATES

## Abstract

**Background:**

Rabies is a serious yet neglected public health threat in resource-limited communities in Africa, where the virus is maintained in populations of owned, free-roaming domestic dogs. Rabies elimination can be achieved through the mass vaccination of dogs, but maintaining the critical threshold of vaccination coverage for herd immunity in these populations is hampered by their rapid turnover. Knowledge of the population dynamics of free-roaming dog populations can inform effective planning and implementation of mass dog vaccination campaigns to control rabies.

**Methodology/Principal Findings:**

We implemented a health and demographic surveillance system in dogs that monitored the entire owned dog population within a defined geographic area in a community in Mpumalanga Province, South Africa. We quantified demographic rates over a 24-month period, from 1^st^ January 2012 through 1^st^ January 2014, and assessed their implications for rabies control by simulating the decline in vaccination coverage over time. During this period, the population declined by 10%. Annual population growth rates were +18.6% in 2012 and -24.5% in 2013. Crude annual birth rates (per 1,000 dog-years of observation) were 451 in 2012 and 313 in 2013. Crude annual death rates were 406 in 2012 and 568 in 2013. Females suffered a significantly higher mortality rate in 2013 than males (mortality rate ratio [MRR] = 1.54, 95% CI = 1.28–1.85). In the age class 0–3 months, the mortality rate of dogs vaccinated against rabies was significantly lower than that of unvaccinated dogs (2012: MRR = 0.11, 95% CI = 0.05–0.21; 2013: MRR = 0.31, 95% CI = 0.11–0.69). The results of the simulation showed that achieving a 70% vaccination coverage during annual campaigns would maintain coverage above the critical threshold for at least 12 months.

**Conclusions and Significance:**

Our findings provide an evidence base for the World Health Organization’s empirically-derived target of 70% vaccination coverage during annual campaigns. Achieving this will be effective even in highly dynamic populations with extremely high growth rates and rapid turnover. This increases confidence in the feasibility of dog rabies elimination in Africa through mass vaccination.

## Author Summary

Rabies is a deadly disease caused by a virus that in Africa is maintained in populations of owned, free-roaming domestic dogs. Rabies can be controlled by mass vaccination, by ensuring that a certain proportion of the dog population is immune to the disease. Maintaining this proportion of immune animals creates herd immunity, reducing the spread of disease even among non-immune individuals, eventually leading to its elimination from the population. Maintaining herd immunity to rabies in free-roaming dog populations can be challenging, particularly in communities that lack regular access to veterinary services. In these communities, mass vaccination is usually implemented in annual campaigns, of relatively short duration. Between campaigns, the proportion of immune individuals in the population declines, often dropping below the critical threshold as vaccinated dogs die and susceptible dogs enter the population through birth or migration. We measured these rates of birth, death and migration in a typical population of free-roaming dogs in South Africa, and showed that vaccinating 70% of the population during annual campaigns would be sufficient to maintain herd immunity to rabies in the period between campaigns. This is achievable even in populations that have high turnover and are growing rapidly—the most challenging circumstances to maintaining herd immunity. These findings increase confidence in the feasibility of eliminating dog rabies from Africa through mass vaccination.

## Introduction

Rabies is a serious yet neglected public health threat in resource-limited communities in sub-Saharan Africa [1]. In these settings, the virus that causes this deadly disease is largely maintained in populations of free-roaming domestic dogs, and is transmitted to people through bites or other contact with the saliva of infectious rabid dogs. Rabies in dog populations (and consequently in humans) can be controlled and in certain circumstances eliminated through the mass vaccination of dogs against the virus [2]. The control of an infectious disease through vaccination relies on vaccinating a sufficient proportion of the host population to effect herd immunity: the phenomenon whereby the risk of infection among susceptible individuals in a population is reduced by the presence of immune individuals [3]. Thus, if a threshold proportion of individuals in a population are immune, the incidence of infection in that population will decline, eventually to zero [3]. This critical vaccination threshold is a function of the basic reproductive number *R*_*0*_; that is, the number of secondary cases of infection generated by a typical infectious individual in an otherwise fully susceptible population [4]. Hampson et al. [5] estimated *R*_*0*_ for outbreaks of rabies in domestic dog populations around the world. From their estimates (*R*_*0*_ < 2), they calculated the critical vaccination threshold for rabies to be lower than 40% in the populations reviewed. Thus, theory and empirical evidence predicts that outbreaks of rabies in dogs can be controlled if at least 40% of the population is immune at any time. However, achieving this goal in free-roaming dog populations in resource-limited communities is hampered by the rapid turnover of these populations, compounded by the lack of affordable and accessible veterinary services. In these areas, mass dog vaccination against rabies is usually implemented by the state or other agencies in annual or less frequent campaigns, of relatively short duration. Between campaigns, the proportion of immune individuals in the population declines as vaccinated dogs die and susceptible dogs enter the population through birth or migration. To maintain population immunity above the critical threshold in the period between campaigns requires that a larger proportion of the dog population be vaccinated during campaigns [5]. The actual target vaccination coverage to be achieved during campaigns is thus dependent on the demographic rates of the dog population, as well as the interval between campaigns and the duration of vaccine-induced immunity.

The World Health Organization (WHO) recommends that, to achieve control and eventual elimination of dog rabies, programmes must ensure that mass dog vaccination campaigns achieve a vaccination coverage of at least 70% of the population in a given area, and that such campaigns recur, usually annually [6]. The figure of 70% is an empirically-derived consensus, stemming from work on the control of dog rabies in New York State during the 1940s [7]. It is assumed that this coverage, achieved during a campaign of relatively short duration, is sufficient to maintain the population immunity above the critical threshold for at least 12 months, despite dog population turnover due to births, deaths and migrations during this period [6]. To date, little work has been done to test this assumption using real data on demographic rates from free-roaming dog populations in rabies-affected communities. Using demographic characteristics for a cohort of owned dogs over a 12-month period in northwest Tanzania, retrospectively collected through household questionnaires, Hampson et al. [5] estimate that a target vaccination coverage of 60% is sufficient to avoid coverage falling below the critical threshold of 40% between annual campaigns. Using prospective cohort studies in four populations of owned, free-roaming dogs in South African and Bali, Indonesia, Morters et al. [8] estimate that 60–70% coverage is sufficient for the same purpose.

Knowledge of the population dynamics of free-roaming dog populations, particularly the core demographic rates of birth, death and migration, may therefore help to inform effective planning and implementation of mass dog vaccination campaigns to control rabies in resource-limited communities, and to design strategies for the eventual elimination of dog rabies and associated human deaths. Knowledge of these rates, and their interplay with population vaccination coverage levels, may also improve understanding of the possible contribution of humane dog population management to rabies control efforts [9, 10]. Despite the ubiquity of free-roaming dogs in sub-Saharan Africa, little is known about the demographic rates of these populations, or the factors that affect them. Studies that have provided estimates of demographic parameters of these populations have largely relied on cross-sectional household questionnaire surveys of dog owners [5, 11–13]. These analyses make certain assumptions which may not hold true, such as stable age- and sex-distributions or consistency of demographic rates over time. In addition, human-mediated migration of domestic dogs may play an important role in population dynamics and rabies epidemiology [11, 12, 14–18], yet few studies have examined the contribution of migration to population turnover.

A number of studies of free-roaming dog populations in communities in Africa have revealed that, despite appearances, there is little evidence for the presence of large numbers of unowned dogs in these populations [11, 12, 16–19]. Adequate demographic surveillance of dog populations is therefore possible through on-going monitoring of owned dogs within households [8]. Here, we report data from a demographic surveillance system covering all owned dogs in a rabies-affected, resource-limited community in South Africa. Data span a 24-month period, from 1^st^ January 2012 through 1^st^ January 2014. The aim of the study was to quantify demographic parameters in this population of dogs, particularly the core demographic rates of births, deaths and migrations, and to assess the implications of dog population dynamics for rabies control through mass vaccination.

## Methods

### Health and demographic surveillance system in dogs (HDSS-Dogs)

The data collection method used in our study follows the model of health and demographic systems in human public health. A health and demographic system (HDSS) monitors all individuals, households and residential units in a defined geographic area, known as a demographic surveillance area (DSA) (www.indepth-network.org). HDSSs are used in the field of public health, to meet the need for reliable population-based data on health in many low- and middle-income countries where there is limited registration of vital events, including births, deaths (by age and sex), and medical causes of death [20]. The overall objective of these HDSS sites is to establish a reliable information base to help policy-makers set health priorities and allocate resources more efficiently. Following an initial census of the defined population in which all residential units and occupants are enumerated, longitudinal measurement of demographic and health variables is undertaken through repeated visits at regular intervals to all residential units within the DSA. We applied this model to create a health and demographic surveillance system in dogs (HDSS-Dogs) in a population of owned, largely free-roaming dogs in a resource-limited community in South Africa. The aim of the HDSS-Dogs is to provide long-term, reliable, population-based data for evidence-based approaches to the control and elimination of dog rabies, and for humane dog population management in resource-limited communities.

### Study area

**Fig 1:**
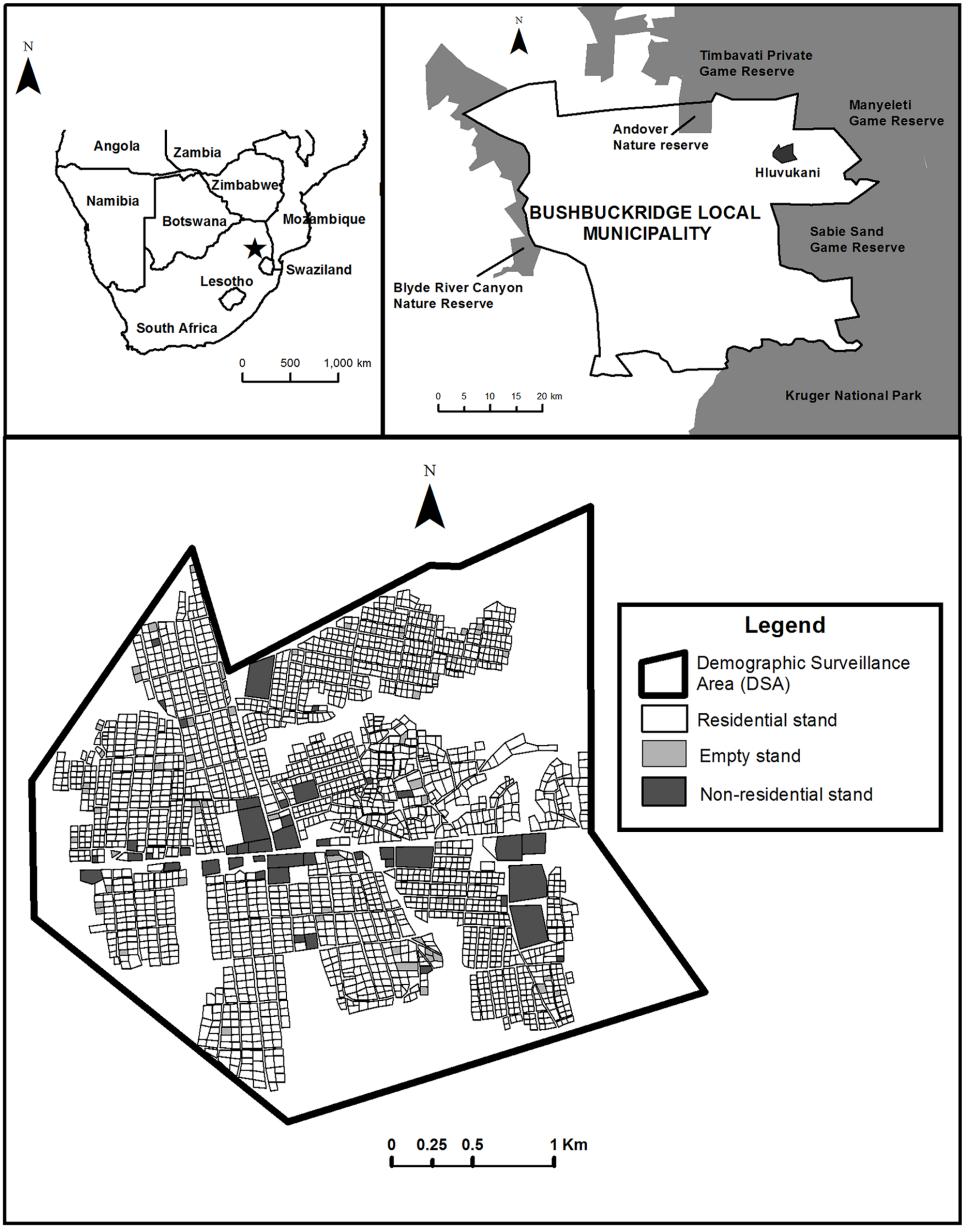
Location of the owned dog demographic surveillance area (DSA) in Hluvukani, South Africa, and of all stands within the DSA.

The DSA of the HDSS-Dogs (Fig 1) was arbitrarily defined prior to the start of the study, making use of natural and man-made features recognisable on the ground by field teams. The DSA encompasses Hluvukani settlement (S 24°39’; E 31°20’), which includes parts of two administrative areas (Eglington and Clare A villages) in Bushbuckridge Local Municipality, Mpumalanga Province, South Africa. The total human population of Bushbuckridge Municipality in 2011 was 541,249, with a growth rate of 0.79% from 2001–2011 [21]. Over two thirds of the population aged 20 years and older had not completed secondary school. The unemployment rate in Bushbuckridge in 2011 was 52.6%, substantially higher than the national rate of 29.8%. The majority of the population (96%) lives in formal housing, with a mean household size of 4.0 persons. One fifth of households do not have access to piped water, and fewer than 10% to refuse removal services. The mean annual household income in 2011 was ZAR 36,569 (about US$ 5,000), less than half of the mean annual income for the province as a whole [21].

In Hluvukani, families live in houses on separate stands (a stand is a plot or parcel of land). Stands are permanently identified by municipal stand numbers that are unique within each administrative area. Stands without municipal numbers were assigned a unique number by the study team. All stands in the DSA are georeferenced as part of the study. There are no private veterinary services in the study area. The provincial state veterinary services have strengthened regular dog rabies vaccination campaigns since the disease re-emerged in Bushbuckridge in 2008, after a long period of apparent absence [22]. In addition to the veterinary services provided by the state, the animal health needs of the community in Hluvukani and the surrounding areas are also met at a subsidised rate by the Hluvukani Animal Clinic, located in the centre of Hluvukani and run by the Faculty of Veterinary Science of the University of Pretoria (UP). The majority of dogs in the community have the morphological appearance of the Africanis landrace [23], although there is some phenotypic evidence of interbreeding with western breeds.

### Data collection

An initial census of the dog population was conducted from July through October, 2011. This census was combined with a house-to-house rabies vaccination campaign in conjunction with the provincial veterinary services and the Faculty of Veterinary Science, UP. To uniquely and permanently identify individual dogs, microchips (BackHome BioTec, Virbac RSA) were subcutaneously implanted into dogs present at the start of the study, and into those dogs that entered the population during the study period. Dogs that could not be handled to implant a microchip were assigned a unique identification code. Dogs were also identified by name and appearance. All dogs enrolled in the study were photographed. Following the census (Round 1), five follow-up rounds (Rounds 2–6) were conducted from December 2011 through May 2014, resulting in all households being visited approximately every six months during this period. All households in the DSA were visited during each round. All owned dogs were recorded at each visit, as were the demographic events that occurred in the period between visits, including births, deaths, and migrations into and out of households. Data including sex, age and rabies vaccination status were collected during the census for all dogs and at each round for new dogs. For vaccination history, we used owners’ reports. Although vaccination certificates are issued by the veterinary services, not all owners consistently keep such certificates. Vaccination data of the veterinary services are aggregated at a local administrative level, and are not always readily available for individual dogs. Dogs vaccinated at any point in the preceding 36 months were considered vaccinated [24, 25]. Variables with time units (e.g. age, or time since entry or exit of a dog) were estimated by the owners. Owners were asked to give a lower and upper estimate, in the time unit of their choice (days, weeks, months, years), reflecting the precision of their estimate. The date of the event was assigned as the midpoint of the estimated range, and the range of the estimate converted to days. All ‘residence episodes’ of individual dogs in households were tracked and aggregated to produce the denominator of dog-time in the population. Residence episodes within households begin with birth or in-migration (e.g. purchase or receipt of a new dog), and terminate with death or out-migration (e.g. sale or gifting of dog to another household). Data from 1^st^ January 2012 through 1^st^ January 2014 are presented here. Data are presented in 3-month periods (annual quarters, abbreviated Q). Point data are provided for the first day of each quarter.

### Regression analysis of mortality rates

Mortality rates were determined as the total number of deaths in a defined population during a specified period, divided by the total number of dog-years lived in the same sub-population over the same period. The subpopulations were characterized by three variables: sex (male or female), age class (0–3 months, 4–11 months, 12–23 months, 24–35 months, ≥36 months), and rabies vaccination status (unvaccinated or vaccinated). Time periods were 2012 and 2013. If dogs changed subpopulations over time, they were split in different records; each record is independent as it describes the dog in one or other specific subpopulation. To model mortality rates in the subpopulations across the two periods, we fitted a Poisson regression model in R [26]. For each record, the number of dog-days was calculated and the log (dog-days) included in the regression as an offset. The four variables (sex, age class, vaccination status and year) as well as all possible interactions (two-, three- and four-way) were included in a maximal model. Model simplification was done by stepwise removal of non-significant variables from the maximal model, starting with the highest-order interactions, and examining the resulting change in deviance of the model. Variables whose removal did not result in a significant change in deviance (p>0.05) were dropped from the model. Mortality rate ratios (MRR) and confidence intervals were calculated from the final minimal adequate model.

### Simulation of vaccination coverage

To assess whether a 70% vaccination coverage achieved during annual campaigns is sufficient to maintain population immunity above the critical threshold of 40% for a 12-month period, we simulated two hypothetical vaccination campaigns, one on 1^st^ January 2012 and another on 1^st^ January 2013. We randomly assigned a positive vaccination status to 70% of all dogs present in the population on those dates. The number of these vaccinated dogs still present in the population 12 months later was divided by the total number of dogs in the population on that date, to give the vaccination coverage one year later. The process of random assignment was repeated 1,000 times to produce Monte Carlo estimates of vaccination coverage. Similarly, we assessed the minimum proportion of dogs to be vaccinated during campaigns on the 1^st^ January 2012 and on the 1^st^ January 2013 required to ensure a 40% coverage in the population 12 months after the respective dates.

### Ethics statement

The study was approved by the University of Pretoria Animal Ethics Committee (protocol no. V033-11). Written informed consent was obtained from dog owners to participate in the study. The protocol adhered to the specifications in the South African National Standard (SANS 10386–2008): “The Care and Use of Animals for Scientific Purposes”.

## Results

### Description of demographic parameters

The area of the DSA is 10.4 km^2^. There are a total of 2,373 stands in the DSA, including 68 empty or non-residential stands (Fig 1). Stands occupy an area of 4.6 km^2^. The total number of households in the DSA recorded during Round 6 was 2,116. A number of households occupy more than one stand. As residence episodes of households within the DSA were not tracked for this study, we present household-level data for Round 6 only. The number of occupants in these households was 9,652, with a mean household size of 4.6 (range: 1–27). The mean number of dogs per household in Round 6 was 0.36 (range 0–9), and the number of dogs per 100 people in the DSA was 7.9. The percentage of dog-owning households (DOHHs) was 17%. The proportion of surgically-sterilized dogs in the population was very low (1–1.5%). We recorded the number of new households (taken occupancy within the previous 12 months) in the DSA during Round 6. Of the 2,116 households, only eight were new, with missing data from a further eleven households.

**Fig 2:**
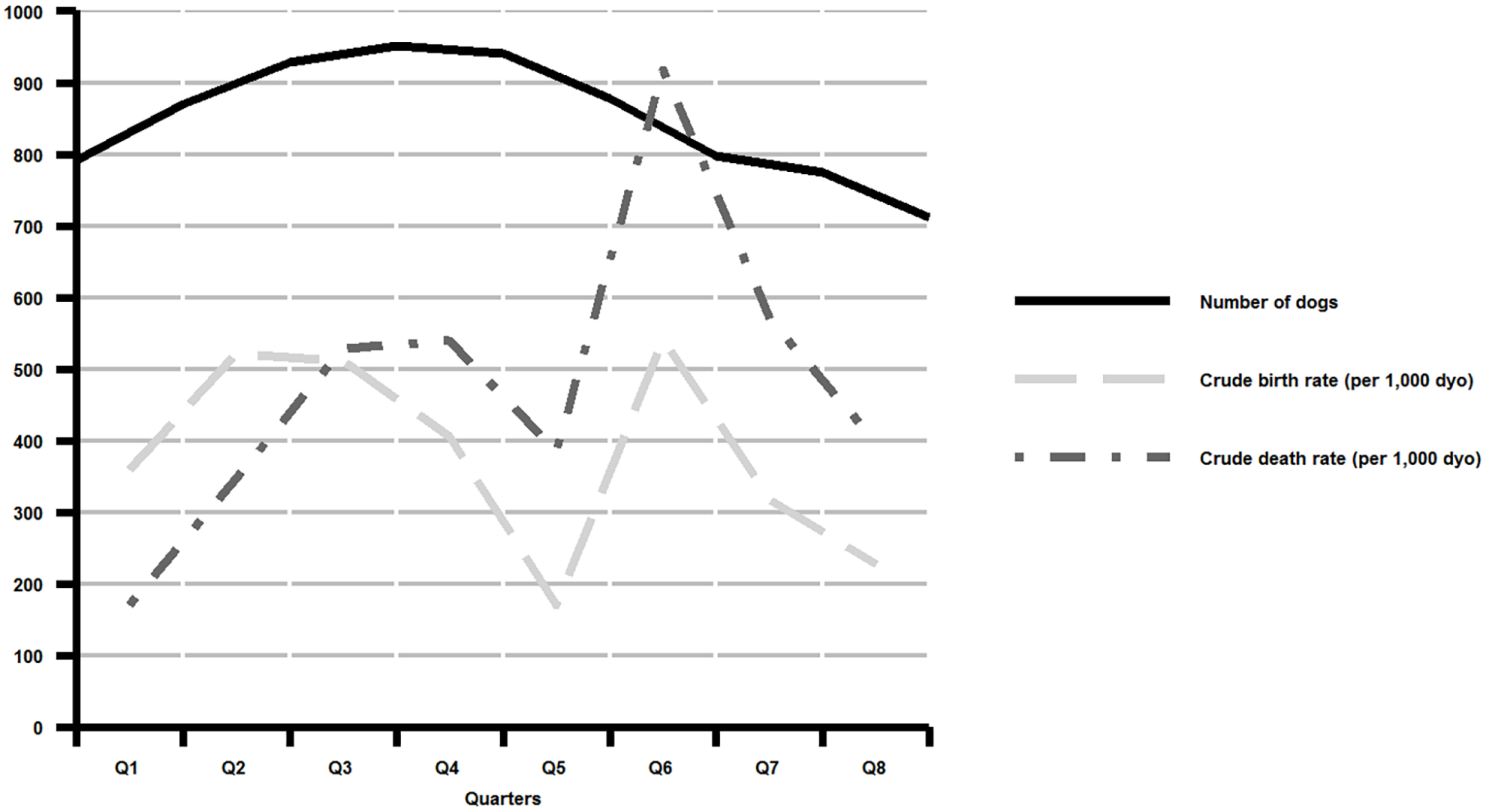
Population size (total number of dogs on the first day of each quarter Q) and crude birth and death rates per 1,000 dog-years of observation (dyo) by quarter, of owned dogs within the demographic surveillance area from 1^st^ January 2012 to 1^st^ January 2014.

Table 1 shows the demographic characteristics of the owned dog population present in the DSA on the first day of each quarter, from 1^st^ January 2012 to 1^st^ January 2014. The population of owned dogs declined by 10% over the period of the study, but this overall decline masks a substantial fluctuation (Fig 2). Annual population growth rate (as a percentage of the population at the start of the period) was +18.6% in 2012 and -24.5% in 2013. The total number of dog-years in the population was 915 in 2012 and 821 in 2013. Crude annual birth rates were 451 puppies born per 1,000 dog-years of observation (dyo) in 2012 and 313 per 1,000 dyo in 2013. Crude annual death rates were 406 per 1,000 dyo in 2012 and 568 per 1,000 dyo in 2013. Crude birth and death rates by quarter are shown in Fig 2. The rate of natural increase of the population (birth rate minus death rate) was +4.5% in 2012 and -25.5% in 2013. The net migration rate, measured as the total number of in-migrations to households minus the total number of out-migrations from households, was 12.3% in 2012 and -2.1% in 2013.

**Table 1:** Demographic characteristics of the owned dog population present in the demographic surveillance area (DSA) on the first day of each quarter, from 1^st^ January 2012 to 1^st^ January 2014.

Year		2012				2013				2014
Quarter		Q1	Q2	Q3	Q4	Q5	Q6	Q7	Q8	Q9
Number of owned dogs		791	870	929	952	941	878	798	775	711
Dog density (km^-2^) in the DSA		76.1	83.7	89.3	91.5	90.5	84.4	76.7	74.5	68.4
Dog-owning households (DOHHs)		396	416	433	449	442	416	393	394	380
Median dogs per DOHH (range)		1 (1–9)	1 (1–13)	1 (1–13)	1 (1–14)	1 (1–13)	1 (1–13)	1 (1–13)	1 (1–18)	1 (1–18)
Sex	Male	456	501	540	554	565	530	489	480	447
	Female	323	357	379	391	375	347	302	282	251
	Unknown	12	12	10	7	1	1	7	13	13
	Sex ratio*	1.41	1.40	1.42	1.42	1.51	1.53	1.62	1.70	1.78
Age (months)	0–3	37	121	136	126	109	38	74	62	52
	4–11	257	178	137	169	192	201	136	108	86
	12–23	187	181	259	263	242	203	157	158	154
	24–35	128	153	159	150	159	144	152	165	157
	≥36	181	236	237	241	236	289	276	279	260
	Unknown	1	1	1	3	3	3	3	3	2

* Males per female

**Fig 3:**
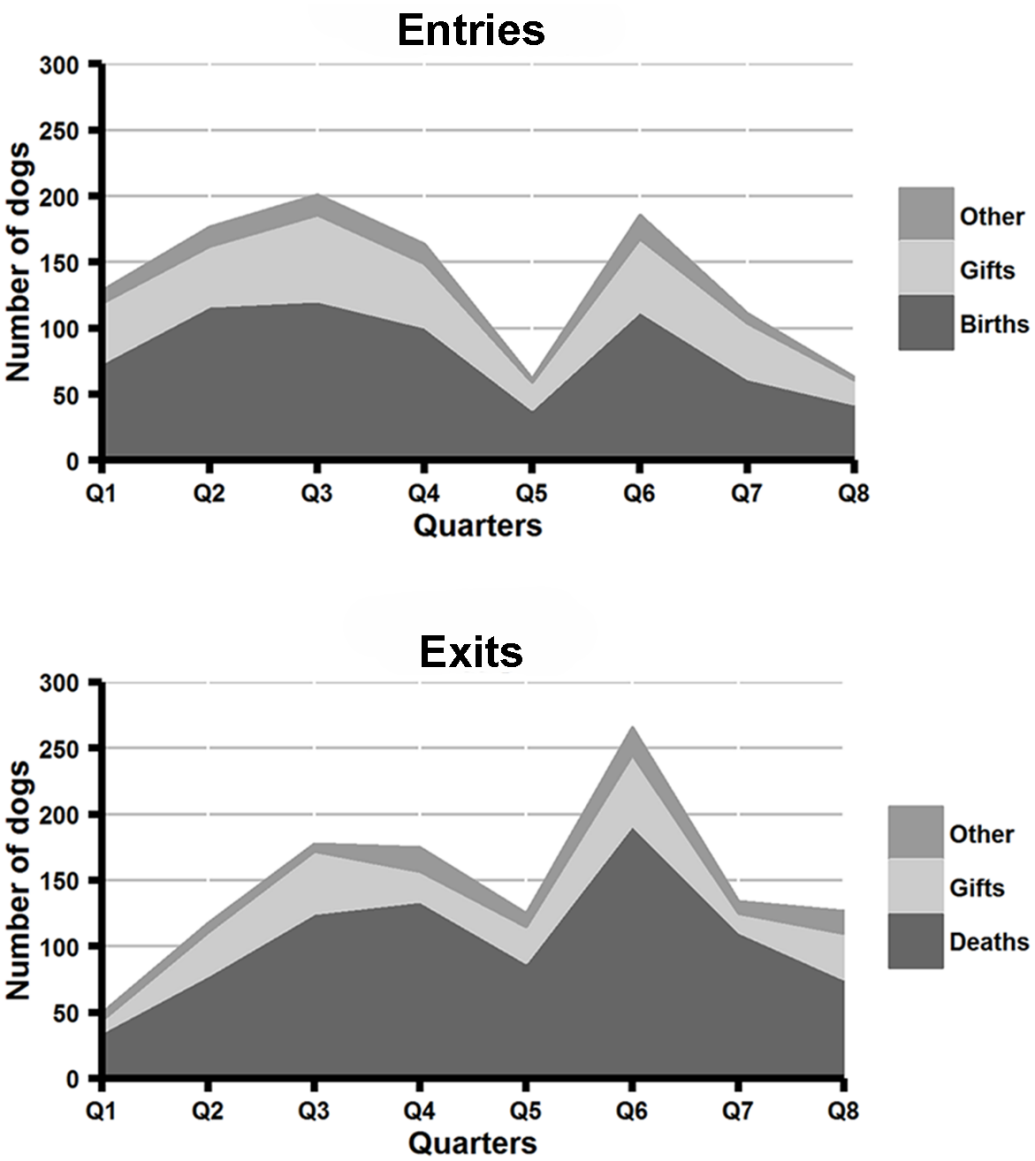
Entry (top panel) and exit (bottom panel) events of owned dogs from households in the demographic surveillance area, from 1^st^ January 2012 to 1^st^ January 2014.

**Fig 4:**
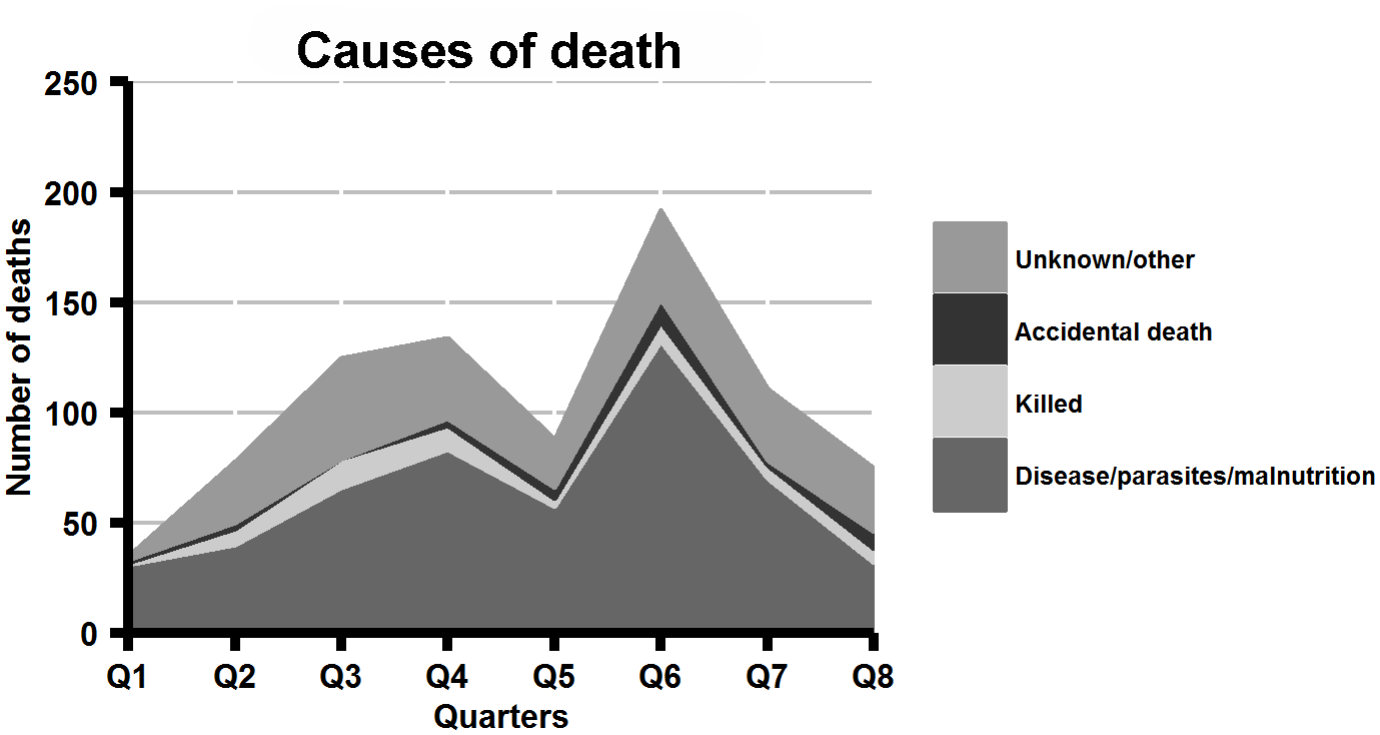
Owner-reported causes of death of owned dogs in the demographic surveillance area, from 1^st^ January 2012 to 1^st^ January 2014.

Data were recorded for 1,093 household entry events and 1,173 exit events (Fig 3). Most dogs entered households through birth (61%) or as gifts (31%), and exited through death (71%) or being given away (21%). Owner-reported causes of death by quarter are shown in Fig 4. Of the exits and entries, a small proportion were dogs that were bought and sold. Over the course of the study, 63 dogs were purchased while only 5 were sold, suggesting that the majority of dogs purchased were from outside the study area. The median purchase price for dogs was ZAR 30, about US$ 5 (n = 60, range: ZAR 5 to ZAR 4,500). The sex ratio of dogs migrating in to the population was skewed towards males (1.79 males per female; data on characteristics of external migrants was collected in Rounds 5 & 6 only). During this period, the sex ratio of dogs entering households from outside the study area (external in-migrants, n = 25) did not differ significantly from those of dogs entering households from within the study area (internal in-migrants, n = 184) (1.79 vs. 1.78; Fisher’s exact test p-value = 1).

### Regression analysis of mortality rates

Mortality rates by sex, age group and vaccination status are shown in Table 2. There was no evidence of overdispersion of the regression model (residual deviance 4453 on 4590 degrees of freedom; goodness-of-fit test p-value = 0.9). Reducing the number of levels for the age class variable from five to three (0–3 months, 4–11 months and ≥12 months) did not cause a significant increase in deviance of the model (*p* = 0.09), and simplified interpretation of the model outputs; age class was therefore reduced to three levels. The initial model revealed a significant three-way interaction between sex, age class and year. To aid interpretation of this interaction, we split the data into two sets by year (2012 and 2013) and modelled these separately. The adjusted mortality rate ratios (MRRs) are presented in Table 3. In 2012, there was no significant difference in mortality rates between the sexes, but in 2013, females suffered a significantly higher mortality rate (MRR = 1.54, 95% CI = 1.28–1.85). Sex-specific mortality rates by quarter are shown in S1 Fig. In both 2012 and 2013, there was a significant two-way interaction between age class and vaccination status (Tables 3 and 4). Among unvaccinated dogs, mortality rates were significantly lower in the 4–11 months and ≥12 months age classes when compared to the 0–3 months age class across both years, but this effect of age on mortality rates was not seen among vaccinated dogs in either year (Table 4). In the age class 0–3 months, the mortality rate of vaccinated dogs was significantly lower than that of unvaccinated dogs (2012: MRR = 0.11, 95% CI = 0.05–0.21; 2013: MRR = 0.31, 95% CI = 0.11–0.69).

**Table 2:** Mortality rates (per 1,000 dog-years of observation) by sex, age group (five levels) and vaccination status for the owned dog population present in the demographic surveillance area, from 1^st^ January 2012 to 1^st^ January 2014.

	2012			2013		
	Dog-years	Deaths	Mortality rate	Dog-years	Deaths	Mortality rate
**Sex**						
Male	555	204	367	521	230	441
Female	381	158	414	326	217	665
**Age group (months)**						
0–3	119	170	1,431	62	108	1,734
4–11	191	53	277	153	111	726
12–23	234	63	269	192	88	458
24–35	159	31	195	158	59	374
≥36	243	56	231	289	99	343
**Vaccination status**						
Unvaccinated	399	247	619	366	278	759
Vaccinated	546	126	230	488	187	383

**Table 3:** Adjusted mortality rate ratios (MRRs) from a multivariate Poisson regression model of sex, age group (three levels) and vaccination status for the owned dog population present in the demographic surveillance area, modelled separately for 2012 and 2013.

	2012			2013		
	Coefficient	MRR (95% CI)	p-value	Coefficient	MRR (95% CI)	p-value
**Sex**						
Male	Not included in final model			Ref.		
Female				0.43	1.54 (1.28–1.85)	<0.001
**Age group (months)**						
0–3	Ref.			Ref.		
4–11	-1.73	0.18 (0.12–0.25)	<0.001	-0.66	0.52 (0.38–0.70)	<0.001
≥12	-2.15	0.12 (0.08–0.16)	<0.001	-1.39	0.25 (0.19–0.33)	<0.001
**Vaccination status**						
Unvaccinated	Ref.			Ref.		
Vaccinated	-2.23	0.11 (0.05–0.21)	<0.001	-1.17	0.31 (0.11–0.69)	0.01
**Interaction term (Vaccination status x Age group)** *						
Vaccination status x Age (4–11 months)	1.57	4.80 (1.84–3.51)	0.002	0.59	1.80 (0.73–5.48)	0.2
Vaccination status x Age (≥12 months)	2.31	10.08 (4.67–5.30)	<0.001	0.97	2.65 (1.14–7.72)	0.04

Ref. = Reference category

*See Table 4 for interpretation of mortality rate ratios for interaction term

**Table 4:** Mortality rate ratios (MRRs) for the interaction term (vaccination status x age group) from the final multivariate Poisson regression model of sex, age group (three levels) and vaccination status for the owned dog population present in the demographic surveillance area, modelled separately for 2012 and 2013. (See Table 3 for coefficients and MRRs of main effects).

		MRR (95% CI)	
		2012	2013
**Vaccination status (reference category: unvaccinated)**			
Vaccinated	0–3 months	0.11 (0.05–0.21)	0.31 (0.11–0.69)
	4–11 months	0.51 (0.08–2.86)	0.56 (0.08–3.79)
	≥12 months	1.08 (0.21–5.35)	0.82 (0.12–5.34)
**Age group (reference category: 0–3 months)**			
4–11 months	Vaccinated	0.85 (0.23–3.35)	0.94 (0.28–3.86)
	Unvaccinated	0.18 (0.12–0.25)	0.52 (0.38–0.70)
≥12 months	Vaccinated	1.17 (0.38–4.07)	0.66 (0.21–2.57)
	Unvaccinated	0.12 (0.08–0.16)	0.25 (0.19–0.33)

### Vaccination coverage

**Fig 5:**
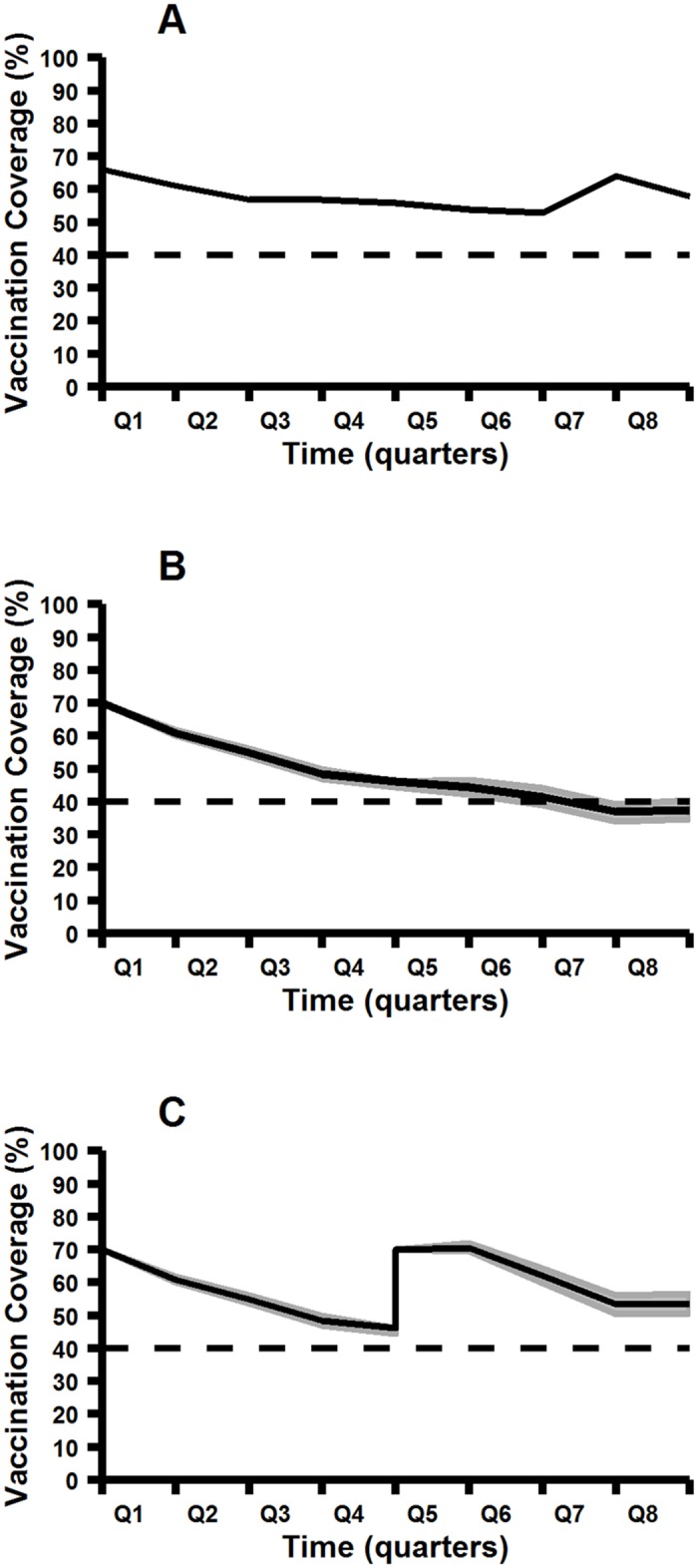
Owner-reported (A) and simulated (B, C) rabies vaccination coverage in owned dogs in the demographic surveillance area, from 1^st^ January 2012 to 1^st^ January 2014. Coverage estimates assume a duration of protective immunity of three years following vaccination. Shaded areas represent minimum/maximum coverage based on 1,000 Monte Carlo simulations. Horizontal dashed line shows the theoretical critical vaccination threshold of 40%. A. Owner-reported vaccination coverage. B. Results of a simulated vaccination campaign on 1^st^ January 2012, reaching a randomly-selected 70% of the dog population present on that date. C. Results of simulated vaccination campaigns on 1^st^ January 2012 and on 1^st^ January 2013, reaching a randomly-selected 70% of the dog population present on those dates.

Vaccination coverage (based on owner-reported vaccination history of 520 dogs for which this information was available) was 33% before the start of the house-to-house vaccination campaign in Round 1. After the campaign, this increased to 78%. Based on owner reports of individual dog vaccination history (and assuming a duration of protection of three years), vaccination coverage remained well above the threshold level of 40% until a second vaccination campaign in 2013 (Fig 5a).

The results of the simulation of vaccination campaigns reaching 70% of the dog population on the 1^st^ January 2012 and the 1^st^ January 2013 are shown in Fig 5b and 5c. Despite the high turnover and substantial growth of the population in 2012, the simulated coverage remained above the threshold of 40% for that year. The decline in the population in 2013 slowed the rate of decrease of vaccination coverage from the 2012 campaign, such that coverage only dropped below the threshold level around 18 months after the campaign (Fig 5b). A second simulated campaign in January 2013 kept coverage well above the threshold for that year (Fig 5c). To ensure a 40% coverage in the population 12 months after vaccination, the minimum vaccination coverage needed for the campaigns was 61% in 2012 and 52% in 2013.

The above simulation assumes that vaccination coverage is randomly distributed in the dog population. We tested for heterogeneity in actual vaccination coverage across sexes and age groups, using owner-reported vaccination data as on 1^st^ January 2012 (coverage = 66%). We found no association between sex and vaccination status (*Χ* ^2^ = 1.02, d.f. = 1, p-value = 0.31), but a strong association between age and vaccination status (*Χ* ^2^ = 71.88, d.f. = 4, p-value < 0.0001), with significantly fewer dogs vaccinated in the 0–3 and 4–11 month age groups than in the oldest age group (≥ 12 months).

## Discussion

We studied the dynamics of an owned, free-roaming dog population over a period of 24 months. We show that this is a highly dynamic population, with rapid turnover and significant heterogeneity in demographic rates over time and across segments of the population. Despite this, routinely achieving 70% vaccination coverage during mass dog vaccination campaigns conducted every 12 months will be sufficient to maintain coverage above the critical threshold of 40%, even during periods of rapid growth and high turnover.

The population declined by 10% over the course of the study. Previous estimates of growth rates of owned dog populations in sub-Saharan Africa predict steady high growth rates of between 7–10% [5, 11–13], but these estimates, derived from retrospective data based on owner recall and collected during household surveys, may not capture the highly dynamic nature of these populations. Moreover, methods used to derive demographic rates from retrospective data collected during cross-sectional surveys may rely on assumptions whose validity is questioned by our findings, such as stable age- and sex-distributions or consistency of demographic rates over time. By contrast, Morters et al. [8], who undertook a longitudinal study of two populations of owned dogs in resource-limited communities in Johannesburg, South Africa using methodologies similar to ours, observed no population growth in one site and an overall decline in the other site, over a three-year period. Together, these longitudinal studies demonstrate that, while owned dog populations are certainly capable of rapid growth, sustained growth in a given area is not a general phenomenon.

The mortality rate in this population of dogs was very high over the study period, particularly in puppies aged 0–3 months. Hampson et al. [5] recorded a similarly high mortality rate (450 deaths per 1,000 population) in dogs older than 3 months in owned, free-roaming dogs in northwest Tanzania. By comparison, the crude death rate of dogs in U.S. households in 1996 was estimated as 79 per 1,000 population [27], and 39 per 1,000 dog-years in insured Swedish dogs from 1995 to 2000 ([28]; this figure excludes puppies and dogs older than 10 years). High mortality rates in the first year of life in free-roaming dogs in resource-limited communities have been reported elsewhere ([11–13, 29]). Over half of all deaths in our study were reported by owners to be due to disease or parasites, while 21% were due to unknown causes. Only a relatively low proportion of deaths (7%) were reportedly caused deliberately by owners or others; however, as Morters et al. (2014) argue, killing of unwanted dogs by owners may be underreported. If it is accepted that human demand rather than environmental resources determines the ‘carrying capacity’ of owned dogs in a given area (as demonstrated by [8]), it can be hypothesized that, in the absence of affordable options for humane population management, killing of dogs will increase as the population grows and demand becomes satiated. Continued observation of the study population through a growth phase may shed further light on this and other mechanisms that regulate population growth.

Mortality rates by age class were highest in the 0–3 month group, and significantly lower in subsequent age classes. Notably, rabies vaccination removed the effect of age on mortality rates, due to its association with significantly reduced mortality rates in the 0–3 month age group. Plausible explanations for this association include i) specific protective immunological effects of the vaccine against rabies in this age group, ii) nonspecific protective immunological effects of the vaccine against other infections (heterologous immunity), and iii) confounding effect of other interventions by owners who have their puppies vaccinated against rabies, compared with those who don’t. It is highly unlikely that the reduction in mortality is due to the specific protective effect of rabies vaccination, given the low incidence of the disease in the population during the periods in question (2012/2013), particularly among the age group concerned [30]. Another explanation might be that dogs that are vaccinated against rabies are simultaneously vaccinated against other infections such as canine distemper or parvovirus, or are more likely to receive therapeutic interventions such as endo- or ectoparasite treatment. The reduction in mortality in the rabies-vaccinated puppies may therefore be as a result of the specific protective or therapeutic effects of associated interventions. Although the overall level of vaccination against diseases other than rabies is assumed to be extremely low, it remains plausible that some owners of young puppies may seek veterinary care at Hluvukani Animal Clinic. Such care might include vaccinations and other healthcare interventions that could significantly increase the survival rates of these puppies. However, cursory examination of clinic records and discussions with the clinician in charge suggest that veterinary health-seeking behaviour among the population within the DSA is not sufficiently advanced to account for this as an explanation. During vaccination campaigns, the local state veterinary services only administer rabies vaccine to dogs (including those in the 0–3 month age class), and no other routine vaccination or healthcare intervention is given. This leaves the intriguing possibility that the reduction in mortality in the 0–3 month age group associated with rabies vaccination is due to a non-specific protective effect of the vaccine. There is strong evidence from human HDSS sites that vaccines have substantial nonspecific effects in children in high-mortality regions [31]; for example, in randomized trials tuberculosis and measles vaccines are associated with a substantial reduction in child mortality, which cannot be explained by prevention of the target disease [32]. Further studies in dogs (observational studies in which the details and timings of vaccinations and other healthcare interventions are carefully recorded, or randomised controlled trials), are needed to determine if rabies vaccine does in fact induce a protective nonspecific immune response sufficient to reduce puppy mortality. Such a finding would have implications for the use of rabies vaccine in this age group (particularly in light of a recent field study showing that rabies vaccine is effective in this age group [33]).

The population sex ratio was strongly skewed towards males (around 1.4 males per female dog in 2012), and became increasingly so during 2013 (increasing from 1.5 to 1.8 males per female). Male-skewed sex ratios are a consistent feature reported in other studies of the demographics of owned dog populations in sub-Saharan Africa [5, 8, 11–13, 19, 34, 35]. This is attributed to a preference of owners for male dogs for guarding of households and livestock, and the reduced nuisance factor of males compared to adult females (e.g. oestrus behaviour, unwanted puppies); however, few studies have directly examined the demographic mechanisms that give rise to this male skew. Preference for male dogs in an owned population implies either decreased retention of female dogs (through higher mortality rates and/or out-migration rates) or increased recruitment of male dogs (through higher in-migration rates or male-skewed birth rates). While Kitala et al. [13] found uniformly lower survival rates for females, other studies in male-skewed populations found no significant differences in survival rates between the sexes [5, 12]. Our study shows that sex-specific mortality rates vary over time, with a significantly higher mortality rate in females compared to males in 2013. This may explain the increase in the sex ratio during that year. (Although the sex ratio of external in-migrants was also male-skewed, the number of external in-migrants was too small to affect the overall population sex ratio). These results highlight the need for longitudinal studies of dog demographics, as contributory factors to population structure will change over time. A conspicuous feature of the mortality rates in this population is the spike in mortality in the second quarter of 2013 (Q6; Fig 2). Although not definitively determined, observations by the authors suggest that a distemper epidemic occurred in the dog population at this time (a retrospective serological study is underway to investigate this hypothesis). Females appear to have suffered a disproportionately greater increase in mortality rates during this period (S1 Fig).

The birth rate in this population was very high (313–451 per 1,000 dog-years), although not sufficient to compensate for the high mortality rates during the latter part of the study period, resulting in the overall natural decline in the population. Hampson et al. [5] report a similar high annual birth rate (530 dogs born per 1,000) for a population of owned, free-roaming dogs in northwest Tanzania. By contrast, New et al. [27] estimated a crude birth rate four times lower (114 dogs born per 1,000) for owned dogs in the United States. Other studies provide proxy measures for birth rates in free-roaming dog populations. Reece et al. [36] report that 48% of roaming adult females became pregnant in any given year from 1995 through 2006 in Jaipur, India. Kitala et al [13] recorded 249 puppies born to a cohort of 305 dogs (128 females and 192 males) over a one-year period in Machakos District, Kenya in 1992–1993, equating to an annual birth rate of 816 dogs born per 1,000 population.

The dynamics of this dog population are strongly seasonal, driven by seasonality in birth rates (peaking April–June) and subsequent mortalities and migration of puppies. Seasonality of breeding in dog populations is not consistently reported. Reece et al. [36] and Totton et al. [37] report seasonal oestrus and pregnancy in two populations of free-roaming dogs in India, while Butler and Bingham [11] deduced a peak in births in June–August in Zimbabwe, neighbouring South Africa to the north. Conversely, Morters et al [8] reported no significant difference in the proportion of dogs pregnant by month in their study populations in Johannesburg, some 350 km south-west of our study area. Seasonality of reproduction, combined with differences in mortality rates across segments of the population, may have implications for the cost-effectiveness of vaccination campaigns conducted at different time periods.

Our simulations show that, despite the highly dynamic nature of this population, achieving 61% vaccination coverage during an annual campaign of relatively short duration will be sufficient to maintain coverage above the critical threshold of 40%, even in the face of rapid growth and high turnover, as was the case in 2012. This is consistent with the predictions of Hampson et al. [5] and Morters et al. [8] from populations of owned, free-roaming dogs in resource-limited communities elsewhere. The predictions from our simulation are conservative, in that they assume that all in-migrating dogs are unvaccinated, and that no supplementary vaccination occurs in the period between campaigns. This may explain the higher-than-predicted estimates seen in the owner-reported vaccination coverage (Fig 5a). Furthermore, the simulation assumed random vaccination of 70% of the dog population present. Because proportionately more puppies were considered vaccinated in our simulation than in reality, and because of the disproportionately higher mortality rate in this age group, our estimates should again be seen as conservative compared to the real-world situation in which a higher proportion of the vaccinated dogs fall within older, more stable age groups. Furthermore, the simulation does not take account of the unanticipated finding in our study, that vaccinated puppies have a significantly lower mortality rate than unvaccinated puppies; this also makes our predictions more conservative. Future refinements of the simulation should take account of heterogeneity in vaccination coverage and demographic rates (particularly mortality rates) across segments of the dog population. Although allowance should be made for the fact that not all dogs who receive the vaccine will develop a protective immune response, the proportion of non-responders is likely small: recent field studies have shown that the vast majority of dogs (>90%) seroconvert to the vaccine, regardless of health status [38].

Overall, the findings of our study are consistent with WHO recommendations that, to achieve control and eventual elimination of dog rabies, programmes must ensure that mass dog vaccination campaigns achieve a vaccination coverage of at least 70% of the population in a given area, and that such campaigns recur, usually annually [6]. Meeting this target ideally requires an accurate estimate of the total dog population in a given area. Obtaining such an estimate is complicated by the highly dynamic nature of dog populations, as evidenced by this study. Estimates based on the number of dogs per household, or per 100 people, should therefore only be used to obtain a rough estimate of dog numbers for planning purposes. Novel, low-cost, robust methods are needed to provide accurate estimates of dog numbers. One such approach may be to engage community members to complete a census of owned dogs immediately ahead of a vaccination campaign. Such an exercise could also be used to raise awareness among dog owners of the upcoming campaign. This approach would be in line with that of the community-directed interventions that have proven successful in the control of other neglected tropical diseases, in which health interventions are undertaken at the community level under the direction of the community itself [39]. This approach could be extended to include planning of the vaccination campaign itself by communities, in partnership with local veterinary services, to ensure maximum vaccination coverage. The incorporation of a simple dog census/household survey into the dog rabies vaccination campaign, as practised in the study area, also offers a direct assessment of the achieved coverage.

Our results show that effective rabies control is possible without adjunct dog population control measures, such as fertility control though sterilization or contraception [10, 40]. One potential benefit of adjunct sterilisation programmes to rabies control could be to reduce population turnover rates and so help sustain vaccination coverage between campaigns, possibly extending the period between campaigns. Extending the period between campaigns (for example, from 12 to 24 months) could result in significant savings in operational costs and reduced ‘vaccinator fatigue’ (a major factor in reduced campaign effectiveness over time; [41]), thereby improving the long-term cost-effectiveness of rabies eradication programmes; however, further cost-benefit studies are needed to weigh this up against the increased cost and time needed for sterilization programmes. Furthermore, the assumption that reducing birth rates through sterilization programmes will result in lower turnover in owned dog populations must be carefully examined. If mortality rates remain high in the face of lowered birth rates, demand for dogs may soon exceed supply, and new dogs may be sourced from outside the population. If not vaccinated, these dogs will contribute to the turnover of the population and offset the benefits of reduced recruitment of unvaccinated puppies. In addition, increased human-mediated in-migration of dogs may foreseeably increase the rate of incursion of rabies [15, 42, 43], complicating eradication efforts. The focus of adjunct population management measures should be to help create stable, healthy, vaccinated populations of dogs; this may require the identification and inclusion of cost-effective interventions to reduce mortality rates as well as birth rates. Such efforts should not detract from the primary goal for rabies control, which is to achieve at least 70% vaccination coverage of the dog population during campaigns.

In conclusion, we emphasise that mass dog vaccination campaigns which reach 70% of the population will be effective in bringing rabies under control and can contribute to rabies elimination, even in populations undergoing extremely high growth rates and rapid turnover. The results of this study demonstrate that demographic surveillance of an entire owned, free-roaming dog population in a resource-limited community in a rabies-affected area is feasible and provides reliable, accurate data that are needed for decision-making. In much the same way that the INDEPTH network has provided reliable population-based data on human health in low-resourced areas [20], we feel there is value in establishing a network of health and demographic surveillance sites where similar methodologies are applied in owned dog populations in resource-limited communities, to provide a platform for evidence-based policies for rabies control and humane dog population management.

## Supporting Information

S1 Fig Sex-specific mortality rates by quarter of owned dogs within the demographic surveillance area from 1^st^ January 2012 to 1^st^ January 2014. Vertical bars show the 95% confidence intervals.
(TIFF)
