# Supplementary figures and images for: Population Dynamics of Owned, Free-Roaming Dogs: Implications for Rabies Control
Source: PLoS Negl Trop Dis. 2015 Nov 6;9(11):e0004177. doi: 10.1371/journal.pntd.0004177 (PMC4636342; doi:10.1371/journal.pntd.0004177)

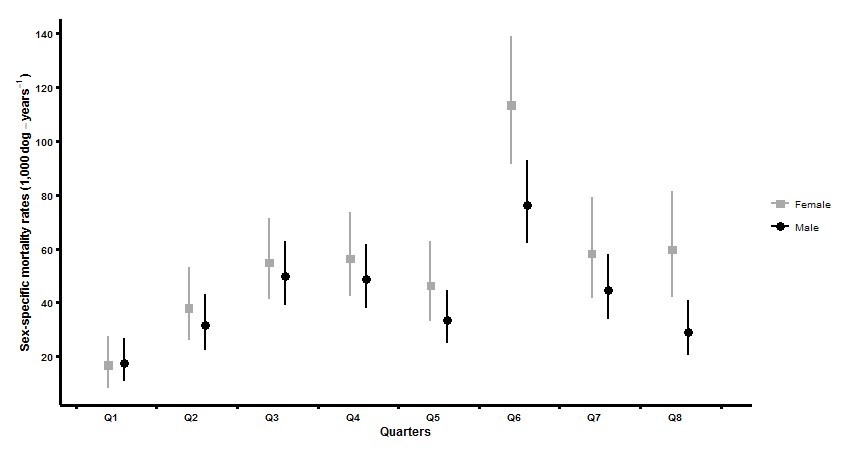

Supplement: S1 Fig — Vertical bars show the 95% confidence intervals. (TIFF) [file pntd.0004177.s001.tiff]
